# Akt activation ameliorates deficits in hippocampal-dependent memory and activity-dependent synaptic protein synthesis in an Alzheimer’s disease mouse model

**DOI:** 10.1016/j.jbc.2023.105619

**Published:** 2024-01-03

**Authors:** Reddy Peera Kommaddi, Ruturaj Gowaikar, Haseena P A, Latha Diwakar, Kunal Singh, Amrita Mondal

**Affiliations:** 1 Centre for Brain Research, Indian Institute of Science, Bangalore, India; 2 Centre for Neuroscience, Indian Institute of Science, Bangalore, India; 3 Manipal Academy of Higher Education, Manipal, India

**Keywords:** activity-dependent protein synthesis, Alzheimer’s disease, APP/PS1, dementia, synaptosomes, synaptic plasticity

## Abstract

Protein kinase-B (Akt) and the mechanistic target of rapamycin (mTOR) signaling pathways are implicated in Alzheimer’s disease (AD) pathology. Akt/mTOR signaling pathways, activated by external inputs, enable new protein synthesis at the synapse and synaptic plasticity. The molecular mechanisms impeding new protein synthesis at the synapse in AD pathogenesis remain elusive. Here, we aimed to understand the molecular mechanisms prior to the manifestation of histopathological hallmarks by characterizing Akt1/mTOR signaling cascades and new protein synthesis in the hippocampus of WT and amyloid precursor protein/presenilin-1 (APP/PS1) male mice. Intriguingly, compared to those in WT mice, we found significant decreases in pAkt1, pGSK3β, pmTOR, pS6 ribosomal protein, and p4E-BP1 levels in both post nuclear supernatant and synaptosomes isolated from the hippocampus of one-month-old (presymptomatic) APP/PS1 mice. In synaptoneurosomes prepared from the hippocampus of presymptomatic APP/PS1 mice, activity-dependent protein synthesis at the synapse was impaired and this deficit was sustained in young adults. In hippocampal neurons from C57BL/6 mice, downregulation of Akt1 precluded synaptic activity–dependent protein synthesis at the dendrites but not in the soma. In three-month-old APP/PS1 mice, Akt activator (SC79) administration restored deficits in memory recall and activity-dependent synaptic protein synthesis. C57BL/6 mice administered with an Akt inhibitor (MK2206) resulted in memory recall deficits compared to those treated with vehicle. We conclude that dysregulation of Akt1/mTOR and its downstream signaling molecules in the hippocampus contribute to memory recall deficits and loss of activity-dependent synaptic protein synthesis. In AD mice, however, Akt activation ameliorates deficits in memory recall and activity-dependent synaptic protein synthesis.

Alzheimer’s disease (AD), the most prevalent form of dementia, is a progressive neurodegenerative disorder associated with the impairment of cognitive functions, including memory (1, 2). Its hallmarks include synapse loss (3, 4, 5, 6, 7), extracellular amyloid-beta peptide (Aβ) plaques, intracellular neurofibrillary tangles, and extensive neurodegeneration (8, 9, 10). Longitudinal studies of AD subjects have consistently shown hyperphosphorylation of tau in plasma and decreased Aβ levels in cerebrospinal fluid and plasma (11, 12, 13, 14). β-amyloid imaging by PET and structural and functional MRI in AD human subjects have revealed increase in β-amyloid levels, decrease in hippocampal volume, and thinning of the cortex preceding the manifestation of clinical and cognitive symptoms by several years or decades (11, 15). Fludeoxyglucose F18-PET imaging revealed lower glucose consumption in AD patients before cognitive impairment (16, 17). However, the early molecular cascades underlying cognitive and synaptic dysfunction in AD are poorly understood.

Akt, an AGC subfamily kinase, regulates cell survival, growth, proliferation, differentiation, metabolism, and insulin-mediated glucose homeostasis (18). The Aktsignaling cascade plays a critical role in neurotransmission and synaptic plasticity (19, 20, 21). Akt requires two phosphorylation events to get completely activated. First, PI3K activation leads to phosphoinositide-dependent kinase-1–mediated phosphorylation of Akt at threonine-308 (Akt-Thr-308), leading to its translocation to the inner leaflet of the plasma membrane from the cytoplasm, where it interacts with phosphatidylinositol (3, 4, 5) trisphosphate and phosphatidylinositol (3, 4) bisphosphate *via* the N-terminal pleckstrin homology domain, modulating a major conformational change. This in turn allows for a second phosphorylation event at serine-473 (Akt-Ser-473) by mTORC2, leading to the full activation of Akt (22).

The mechanistic target of rapamycin (mTOR) signaling regulates cell growth, proliferation, brain development, neural circuit creation, physiology, and metabolism (23). mTOR, a serine/threonine protein kinase (∼289 kDa), forms the catalytic subunit of two functionally distinct mTOR complexes: mTORC1 and mTORC2. While the mTORC1 complex is implicated in neuronal excitability, long-term memory formation (24, 25, 26, 27, 28, 29), and learning (30), the mTORC2 complex is involved in cell migration and cytoskeletal reorganization and integrity (31, 32, 33). However, the majority of neurological diseases are associated with the mTOR cascade through mTORC1 (34). The Akt/mTOR signaling pathway, activated by synaptic activity, modulates local mRNA translation at the synapses (35), and phosphorylation of additional protein substrates (36, 37, 38). Moreover, this pathway regulates neuronal structure and size, spine architecture, and synaptic plasticity (39), affecting both translation initiation-related p70 ribosomal S6 kinases 1 and 2 (S6K1/2) and eukaryotic translation initiation factor 4E-binding proteins (4E-BPs) *via* postsynaptic membrane receptors (40). In a nonphosphorylated state, 4E-BP1 inhibits translation initiation by preventing eukaryotic translation initiation factor 4E–an initiation factor–from recruiting mRNA to the small subunit of ribosomes via the 5′Cap structure of mRNAs. Activated mTOR phosphorylates 4E-BP1 at threonine 46/47 to release eukaryotic translation initiation factor 4E. Thus, activation of the Akt/mTOR signaling cascade is critical at the synapse for new protein synthesis and optimal functioning.

Dysregulation of the Akt/mTOR signaling pathway may lead to neurodegenerative diseases such as AD (21, 28, 41, 42). Studies of postmortem brain tissue obtained from individuals with AD as well as animal models of AD have investigated the disruption of the Akt/mTOR signaling pathway (41, 43). However, the manner in which Akt1 and mTOR signaling pathways regulate baseline and activity-dependent protein synthesis at the synapse in the hippocampus has not been studied in the AD mouse model. The molecular foundations of the hippocampal Akt/mTOR signaling pathway dysregulation and its functional effects on neurodegeneration remain unknown.

We therefore investigated whether the Akt1/mTOR signaling pathway and activity-dependent protein synthesis are perturbed in the hippocampus of a transgenic AD mouse model. The transgenic mouse model chosen for this study was APPswe/PS1ΔE9. These mice express a chimeric mouse/human amyloid precursor protein (Mo/HuAPP695Swe) and mutant human presenilin1 (PS1ΔE9). The accumulation of Aβ plaques begins to emerge in the cortex and hippocampus at about 4 and 6 months of age, respectively (44, 45). Studies have demonstrated the presence of Aβ oligomers prior to Aβ plaque deposition (46), leading to dysregulation of synaptic function, synaptic pruning, loss of dendritic spines, and memory loss (47, 48, 49, 50). The APPswe/PS1ΔE9 mouse model exhibits limitations, including premature death (10%–35%) (51), seizures (44), cardiomyocyte contractile dysfunction (52), hyper excitability (44), and high variability in its mortality rate. Despite its limitations, the APPswe/PS1ΔE9 (hereafter referred to as APP/PS1) mouse model is well established and extensively used in preclinical research to study the molecular mechanisms in AD pathogenesis. Further, it is associated with the early-onset AD and has been used to study the early neuropathological characteristics of AD. We therefore hypothesize that in APP/PS1 mice, soluble Aβ oligomers have the potential to influence activity-dependent protein synthesis by perturbing the Akt/mTOR signaling cascade far in advance of the manifestation of histopathological features.

We conducted the experimental procedures of this investigation well before the occurrence of A-β plaques. We measured activity-dependent protein synthesis and Akt1/mTOR phosphorylation in post nuclear supernatant and synaptosomes from the hippocampus of adolescent and young (1- and 3-month-old, respectively) WT and APP/PS1 male mice. We used shRNA against Akt1 to demonstrate that Akt1 is required for activity-dependent synaptic protein synthesis in primary hippocampal neurons. We found the Akt1/mTOR signaling cascade and activity-dependent protein synthesis at the synapse to be dysregulated in the hippocampus of APP/PS1 mice. In the hippocampal neurons of C57BL/6 mice, depletion of Akt1 expression precluded activity-dependent protein synthesis at the dendrites. Administration of Akt activator (SC79) in APP/PS1 mice restored deficits in both memory recall and activity-dependent synaptic protein synthesis. We conclude that dysregulation of Akt1/mTOR and its downstream signaling molecules in the hippocampus contribute to memory recall deficits and loss of activity-dependent synaptic protein synthesis, which are restored by Akt activation.

## Results

### Activation of Akt1 signaling is diminished in the hippocampus of adolescent APP/PS1 male mice

**Figure 1:**
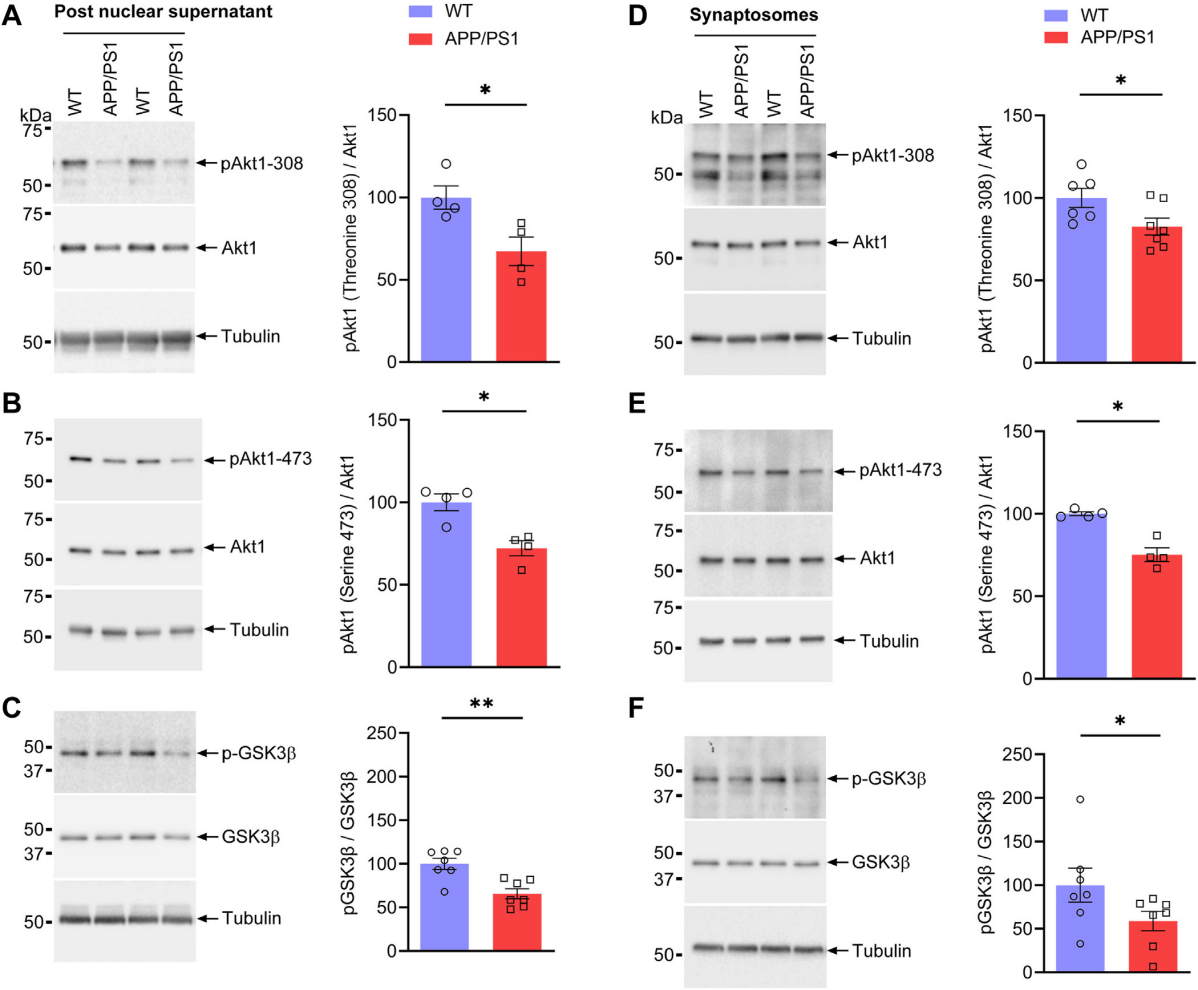
**Akt1 and GSK3β phosphorylation is decreased in post nuclear supernatant and synaptosomes in the hippocampus of APP/PS1 male mice.** Representative immunoblots of (*A*) phosphorylation of Akt1 (threonine-308) and total Akt1 (*p* = 0.0286) (n = 4), (*B*) phosphorylation of Akt1 (serine-473) and total Akt1 (*p* = 0.0286) (n = 4), (*C*) phosphorylation of GSK3β and total GSK3β (*p* = 0.0041) (n = 7) in post nuclear supernatant from the hippocampus of 1-month-old (adolescent) WT and APP/PS1 mice. The representative Western blots for (D) phosphorylation of Akt1 (threonine-308) and total Akt1 (*p* = 0.0350) (n = 6), (*E*) phosphorylation of Akt1 (serine-473) and total Akt1 (*p* = 0.0286) (n = 4), (*F*) phosphorylation of GSK3β and total GSK3β (*p* = 0.0379) (n = 7) in synaptosomes isolated from hippocampus of 1-month-old (adolescent) WT and APP/PS1 mice. All immunoblots were stripped and reprobed for β-tubulin. Densitometric scan analysis depicted as *bar graphs* (*right panels*). Statistical analysis: unpaired, two-tailed Mann–Whitney U test; Data are expressed as mean ± SEM. ∗ (*p* < 0.05), ∗∗ (*p* < 0.01) denotes values significantly different from WT controls. APP/PS1, amyloid precursor protein/presenilin-1.

The Akt1 signaling pathway plays a crucial role in hippocampal synaptic plasticity in the CA1 region and is required for regulating activity-dependent protein synthesis at the synapse during late-phase long-term potentiation (LTP) (20). We therefore designed experiments to understand the molecular mechanisms behind the role of Akt1 signaling in the hippocampus. We evaluated the status of several key molecules of the Akt1 signaling cascade in post nuclear supernatant and synaptosomes isolated from one-month-old WT and APP/PS1 mice. We found the phosphorylation of Akt1 at Thr308 and Ser473 to be significantly decreased in both post nuclear supernatant (Fig. 1, *A* and *B*; pAkt1-Thr308/Akt1, *p* = 0.0286, Hodges–Lehmann estimate: 36.25; pAkt1-Ser-473/Akt1, *p* = 0.0286, Hodges–Lehmann estimate: 27.86) and synaptosomes (Fig. 1, *D* and *E*; pAkt1-Thr308/Akt1, *p* = 0.0350, Hodges–Lehmann estimate: 17.39; pAkt1-Ser-473/Akt1, *p* = 0.0286, Hodges–Lehmann estimate: 25.91) of one-month-old APP/PS1 mice compared with those of WT mice, while this was not affected in post nuclear supernatant of the brain cortex from APP/PS1 mice aged 1 month (Fig. S1, *A* and *B*). Further, the phosphorylation of GSK3β (Ser9)–a downstream effector molecule in Akt1 signaling pathway–was significantly decreased in both post nuclear supernatant (Fig. 1*C*; *p* = 0.0041; Hodges–Lehmann estimate: 34.29) and synaptosomes (Fig. 1*F*; *p* = 0.0379, Hodges–Lehmann estimate: 28.52) of one-month-old APP/PS1 mice compared with that of age matched WT mice.

### mTOR signaling is affected in the hippocampus of adolescent APP/PS1 male mice

**Figure 2:**
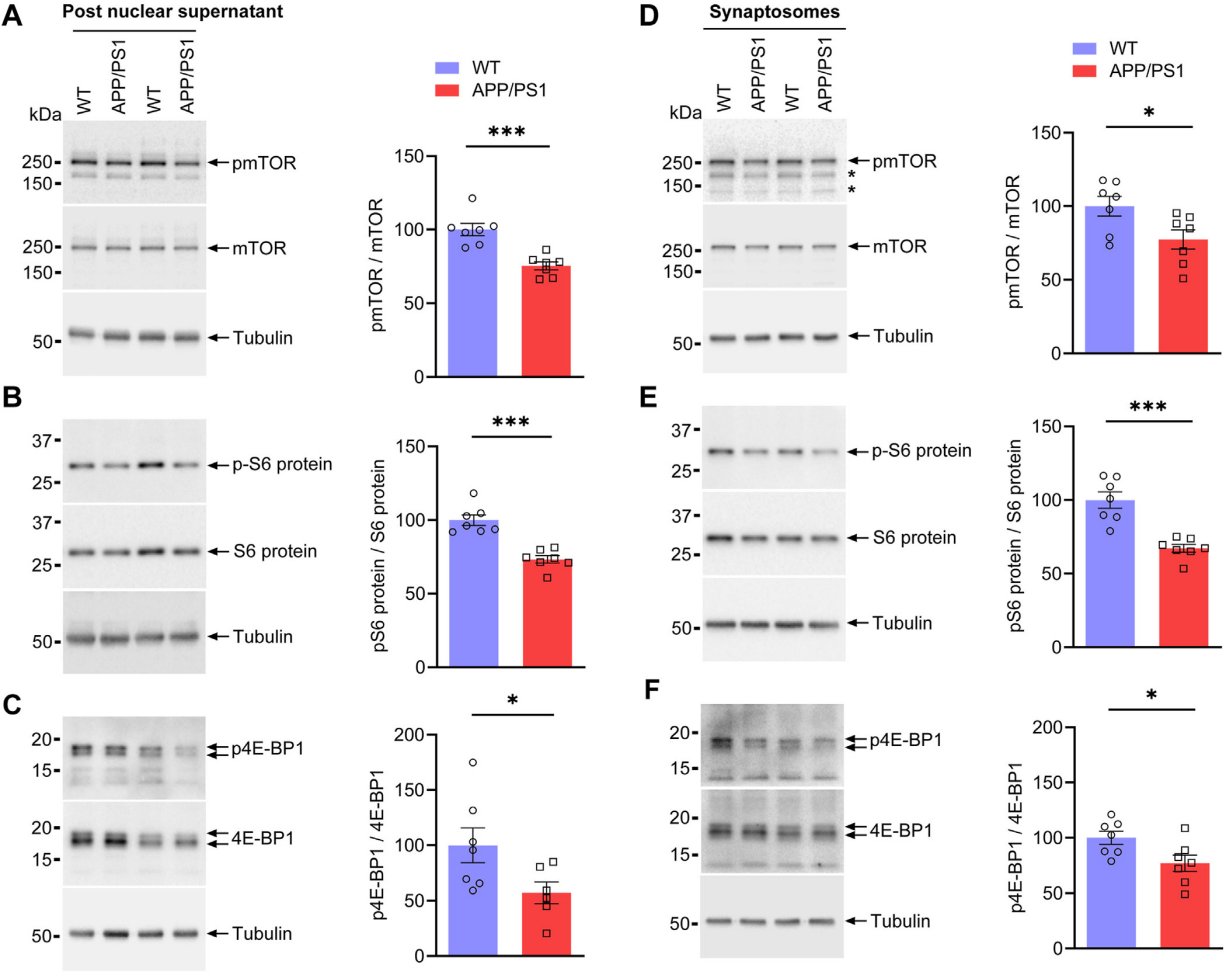
**Mechanistic target of rapamycin signaling cascade proteins are disrupted in post nuclear supernatant and synaptosomes in the hippocampus of APP/PS1 male mice.** Western blots of the mTOR signaling components (*A*) phosphorylation of mTOR and total mTOR (*p* = 0.0006) (n = 7), (*B*) phosphorylation of S6 ribosomal protein and total S6 ribosomal protein (*p* = 0.0006) (n = 7), (*C*) phosphorylation of 4E-BP1 and total 4E-BP1 (*p* = 0.0350) (WT, n = 7; APP/PS1, n = 6) in post nuclear supernatant from the hippocampus of 1-month-old (adolescent) WT and APP/PS1 mice. Representative immunoblots for the mTOR signaling proteins (*D*) phosphorylation of mTOR and total mTOR (*p* = 0.0379) (n = 7), “∗” indicates as nonspecific protein bands, (*E*) phosphorylation of S6 ribosomal protein and total S6 ribosomal protein (*p* = 0.0006) (n = 7), (*F*) phosphorylation of 4E-BP1 and total 4E-BP1 (*p* = 0.0379) (n = 7) in synaptosomes isolated from the hippocampus of 1 month (adolescent) old WT and APP/PS1 mice. All immunoblots were stripped and reprobed for β-tubulin. Quantification of protein band intensities is shown as *bar graphs* (*right panels*). The phosphorylated/total protein levels of each protein were normalized to corresponding WT controls. Statistical analysis: unpaired, two-tailed Mann–Whitney U test; Data are depicted as mean ± SEM. ∗ (*p* < 0.05), ∗∗∗ (*p* < 0.001) denotes values significantly different from WT controls. 4E-BP, 4E-binding protein; APP/PS1, amyloid precursor protein/presenilin-1; mTOR, mechanistic target of rapamycin.

mTOR is an essential effector molecule of the Akt signaling pathway regulating activity-dependent protein synthesis through the activation of S6 ribosomal protein and 4E-BP1. Hence, we attempted to determine whether mTOR signaling was perturbed in the hippocampus during early AD pathogenesis. We isolated post nuclear supernatant and synaptosomes from the hippocampus of one-month-old WT and APP/PS1 mice to examine the protein levels of mTOR and its downstream signaling proteins. We found the phosphorylation of mTOR (serine-2448) to be significantly decreased in both post nuclear supernatant (Fig. 2*A*; *p* = 0.0006, Hodges–Lehmann estimate: 22.83) and synaptosomes (Fig. 2*D*; *p* = 0.0379, Hodges–Lehmann estimate: 22.43) of one-month-old APP/PS1 mice compared to that of age matched WT mice, unlike that in the post nuclear supernatant of the brain cortex in APP/PS1 mice (Fig. S2*A*). Further, we investigated the phosphorylation of S6 ribosomal protein (Ser240/244) and 4E-BP1 (Thr37/Thr46), detecting that the phosphorylation of both the former (Fig. 2, *B* and *E*) and latter (Fig. 2, *C* and *F*) were significantly diminished in both post nuclear supernatant (Fig. 2, *B* and *C*; pS6 protein/S6 protein, *p* = 0.0006, Hodges–Lehmann estimate: 23.20; p4EBP1/4EBP1, *p* = 0.0350, Hodges–Lehmann estimate: 41.07) and synaptosomes (Fig. 2, *E* and *F*; pS6 protein/S6 protein, *p* = 0.0006, Hodges–Lehmann estimate: 34.71; p4EBP1/4EBP1, *p* = 0.0379, Hodges–Lehmann estimate: 25.14) of one-month-old APP/PS1 mice compared with those of WT mice.

### Activity-dependent protein synthesis is impaired in the hippocampus of APP/PS1 male mice

**Figure 3:**
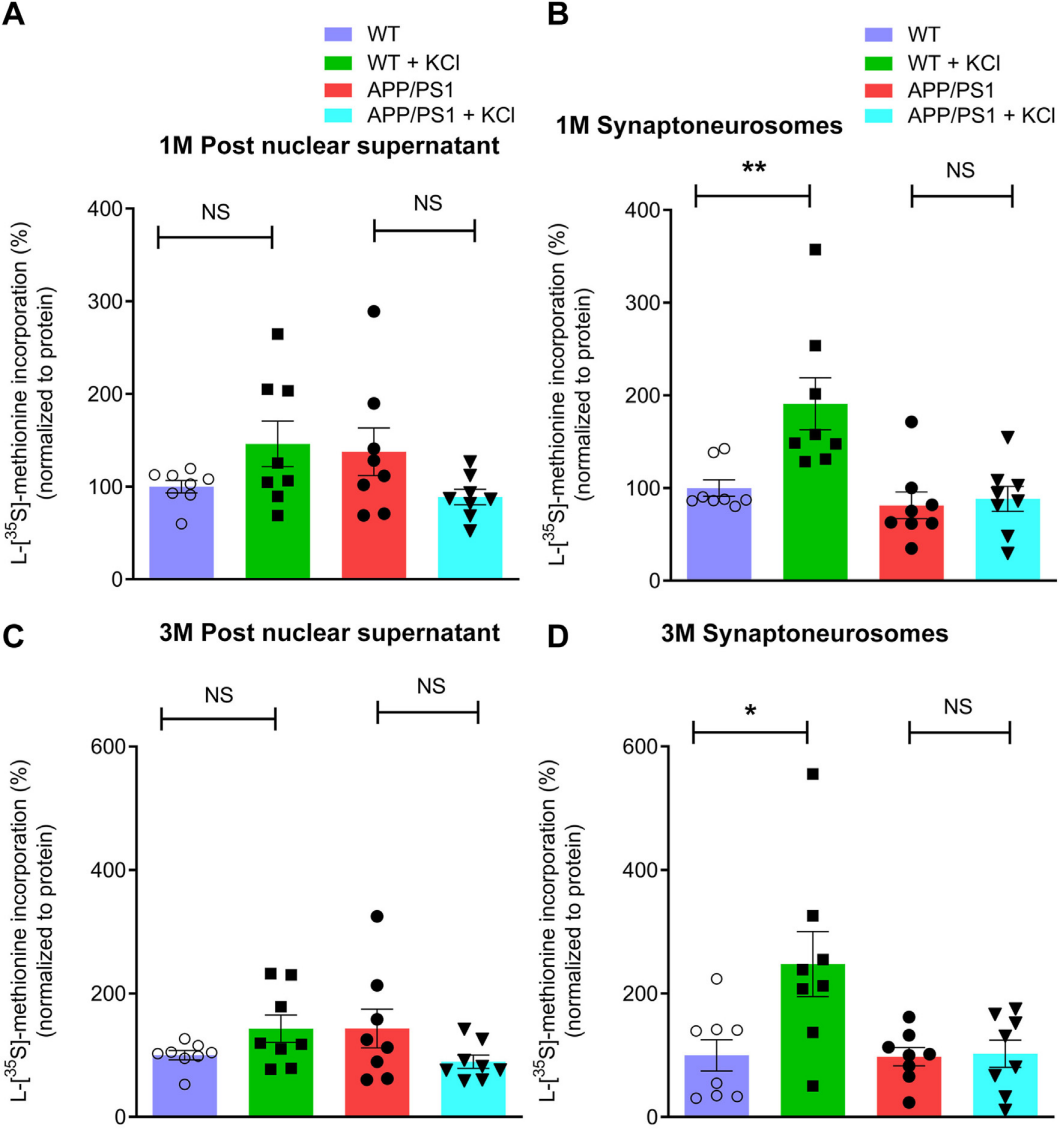
**Activity-dependent protein synthesis at the synapse is deficient in the hippocampus of APP/PS1 male mice.** Activity-dependent protein synthesis was measured by [^35^S]-L-methionine incorporation using post nuclear supernatant and synaptoneurosomes isolated from the hippocampus of WT and APP/PS1 mice. Representative *bar graphs* were showing the quantity of [^35^S]-L-methionine incorporation during new protein synthesis at the synapse in the absence or presence of KCl. Activity-dependent protein synthesis by KCl stimulation in post nuclear supernatant was not affected in (*A*) 1-month and (*C*) 3-month-old APP/PS1 mice compared with age-matched WT mice (1 month post nuclear supernatant, WT *versus* WT + KCl, *p* = 0.3282; APP/PS1 *versus* APP/PS1 + KCl, *p* = 0.1049) (3 months post nuclear supernatant, WT *versus* WT + KCl, *p* = 0.1605; APP/PS1 *versus* APP/PS1 + KCl, *p* = 0.1949). Activity-dependent protein synthesis in synaptoneurosomes in the presence of KCl was absent in (*B*), 1-month and (*D*), 3-month-old APP/PS1 mice compared with age-matched WT mice (1 month synaptoneurosomes, WT *versus* WT + KCl, *p* = 0.0019; APP/PS1 *versus* APP/PS1 + KCl, *p* = 0.5054) (3 months synaptoneurosomes, WT *versus* WT + KCl, *p* = 0.0281; APP/PS1 *versus* APP/PS1 + KCl, *p* = 0.8785). Statistical analysis: unpaired, two-tailed Mann–Whitney U test; data are represented as mean ± SEM. (n = 8 mice). ∗ (*p* < 0.05), ∗∗ (*p* < 0.01) denotes values significantly different from WT *versus* WT + KCl or APP/PS1 *versus* APP/PS1 + KCl. APP/PS1, amyloid precursor protein/presenilin-1.

Activity-dependent protein synthesis mediated by Akt/mTOR signaling pathway at the synapse is an essential event for synaptic plasticity and synaptic function. It is impaired in synaptoneurosomes isolated from the brain cortex of adolescent and young APP/PS1 mice (43). Here, we sought to determine whether activity-dependent protein synthesis at the synapse is affected by KCl stimulation in post nuclear supernatant and synaptoneurosomes in the hippocampus of adolescent and young APP/PS1 mice. We found that upon KCl stimulation, adolescent and young (1 and 3 months of age, respectively) WT mice showed a significant increase in protein synthesis in synaptoneurosomes (1 month of age, *p* = 0.0019; 3 months of age, *p* = 0.0281) but not in post nuclear supernatant (1 month of age, *p* = 0.3282; 3 months of age, *p* = 0.1605) (Fig. 3, *A*–*D*). However, in age-matched adolescent and young APP/PS1 mice, we did not observe protein synthesis by KCl stimulation in both post nuclear supernatant (1 month of age, *p* = 0.1049, Hodges–Lehmann estimate: 31.40; 3 months of age, *p* = 0.1949) and synaptoneurosomes (1 month of age, *p* = 0.5054, Hodges–Lehmann estimate: 15.86; 3 months of age, *p* = 0.8785) (Fig. 3, *A*–*D*). Our results thus provide evidence that activity-dependent protein synthesis at the synapse in the hippocampus of APP/PS1 mice is affected at as early as 1 month of age.

### Depletion of Akt1 expression abolishes activity-dependent protein synthesis in neurites

**Figure 4:**
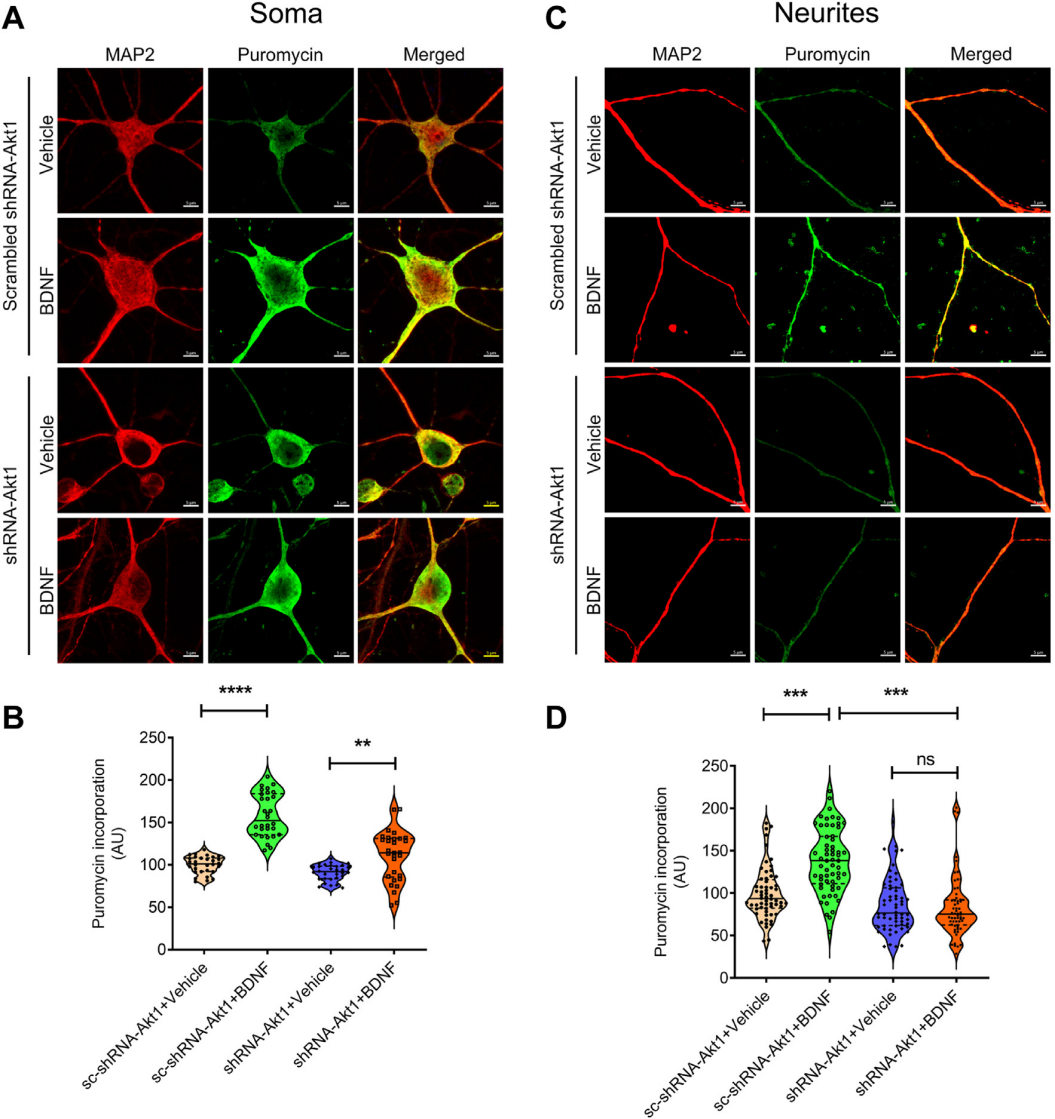
**Downregulation of Akt1 abolishes BDNF-stimulated protein synthesis in tertiary neurites of hippocampal neurons.** Primary hippocampal neurons were infected with a control lentivirus (scrambled shRNA-Akt1) or a lentivirus encoding shRNA against mouse Akt1 (shRNA-Akt1) at *DIV18*. Neurons were immunostained with anti-puromycin antibody and coimmunostained for the neuronal marker microtubule associated protein 2 (MAP2). MAP2 is a dendritically enriched general protein marker, while puromycin is used to detect newly synthesized proteins. *A*, representative confocal images of cell body (soma) of primary hippocampal neurons at *DIV21* are showing the immunostaining signal for MAP2 (*red*) and puromycin (*green*), respectively. The scale bar represents 5 μm. *B*, *violin plot* showing the quantification of puromycin incorporation as green signal intensity. Data are represented as mean ± SEM from three independent experiments. n = 10 neurons from each independent experiment. Statistical significance indicated as ∗∗*p* < 0.01, ∗∗∗*p* < 0.001, and two-way ANOVA, followed by Tukey’s post test. Interaction: F (1, 116) =26.17, *p* < 0.0001; BDNF: F (1, 116) =107.8, *p* < 0.0001; shRNA: F (1, 116) =56.69, *p* < 0.0001). (sc-shRNA-Akt1+vehicle *versus* shRNA-Akt1+BDNF; *p* = 0.3248; sc-shRNA-Akt1+vehicle *versus* sc-shRNA-Akt1+BDNF, *p* < 0.0001; sc-shRNA-Akt1+vehicle *versus* shRNA-Akt1+BDNF, *p* = 0.1869; shRNA-Akt1+vehicle *versus* sc-shRNA-Akt1+BDNF, *p* < 0.0001; shRNA-Akt1+vehicle *versus* shRNA-Akt1+BDNF, *p* = 0.0017; sc-shRNA-Akt1+BDNF *versus* shRNA-Akt1+BDNF, *p* < 0.0001). *C*, representative confocal images of tertiary neurites of primary hippocampal neurons at *DIV21* are showing the immunostaining signal for MAP2 (*red*) and puromycin (*green*), respectively. The scale bar represents 5 μm. *D*, *violin plot* showing the quantification of puromycin incorporation as *green* signal intensity. Data are represented as mean ± SEM from three independent experiments. n = 10 neurons and 20 tertiary neurites (two neurites from each neuron) from each independent experiment. Statistical significance indicated as ∗∗∗*p* < 0.001 and two-way ANOVA followed by Tukey’s post test. Interaction: F (1, 236) =21.30, *p* < 0.0001; BDNF: F (1, 236) =15.95, *p* < 0.0001; shRNA: F (1, 236) =60.39, *p* < 0.0001). (sc-shRNA-Akt1+vehicle *versus* sc-shRNA-Akt1+BDNF, *p* < 0.0001; sc-shRNA-Akt1+vehicle *versus* shRNA-Akt1+vehicle; *p* = 0.1177; sc-shRNA-Akt1+vehicle *versus* shRNA-Akt1+BDNF, *p* = 0.0401; shRNA-Akt1+vehicle *versus* sc-shRNA-Akt1+BDNF, *p* < 0.0001; shRNA-Akt1+vehicle *versus* shRNA-Akt1+BDNF, *p* = 0.9716; sc-shRNA-Akt1+BDNF *versus* shRNA-Akt1+BDNF, *p* < 0.0001). APP/PS1, amyloid precursor protein/presenilin-1; BDNF, brain-derived neurotrophic factor.

The Akt/mTOR signaling cascade plays a critical role in activity-dependent protein synthesis at the synapse in neurons. To determine the role of Akt1 on activity-dependent protein synthesis, we utilized the loss-of-function approach by downregulation of Akt1 through shRNA (shAkt1) in primary hippocampal neurons. We found that downregulation of this had no impact on brain-derived neurotrophic factor (BDNF)-stimulated protein synthesis at the soma (Fig. 4, *A* and *B*) (Fig. 4*B*, interaction: F (1, 116) = 26.17, *p* < 0.0001; BDNF: F (1, 116) = 107.8, *p* < 0.0001; shRNA: F (1, 116) = 56.69, *p* < 0.0001). However, when we measured the BDNF-stimulated protein synthesis in tertiary neurites, a proxy for synaptic mRNA translation, we found that the downregulation of Akt1 had a negative impact on activity-dependent protein synthesis (Fig. 4, *C* and *D*) (Fig. 4*D*, interaction: F (1, 236) = 21.30, *p* < 0.0001; BDNF: F (1, 236) = 15.95, *p* < 0.0001; shRNA: F (1, 236) = 60.39, *p* < 0.0001).

### Effect of SC79 and MK2206 on contextual fear memory and activity-dependent synaptic protein synthesis in the hippocampus of male mice

**Figure 5:**
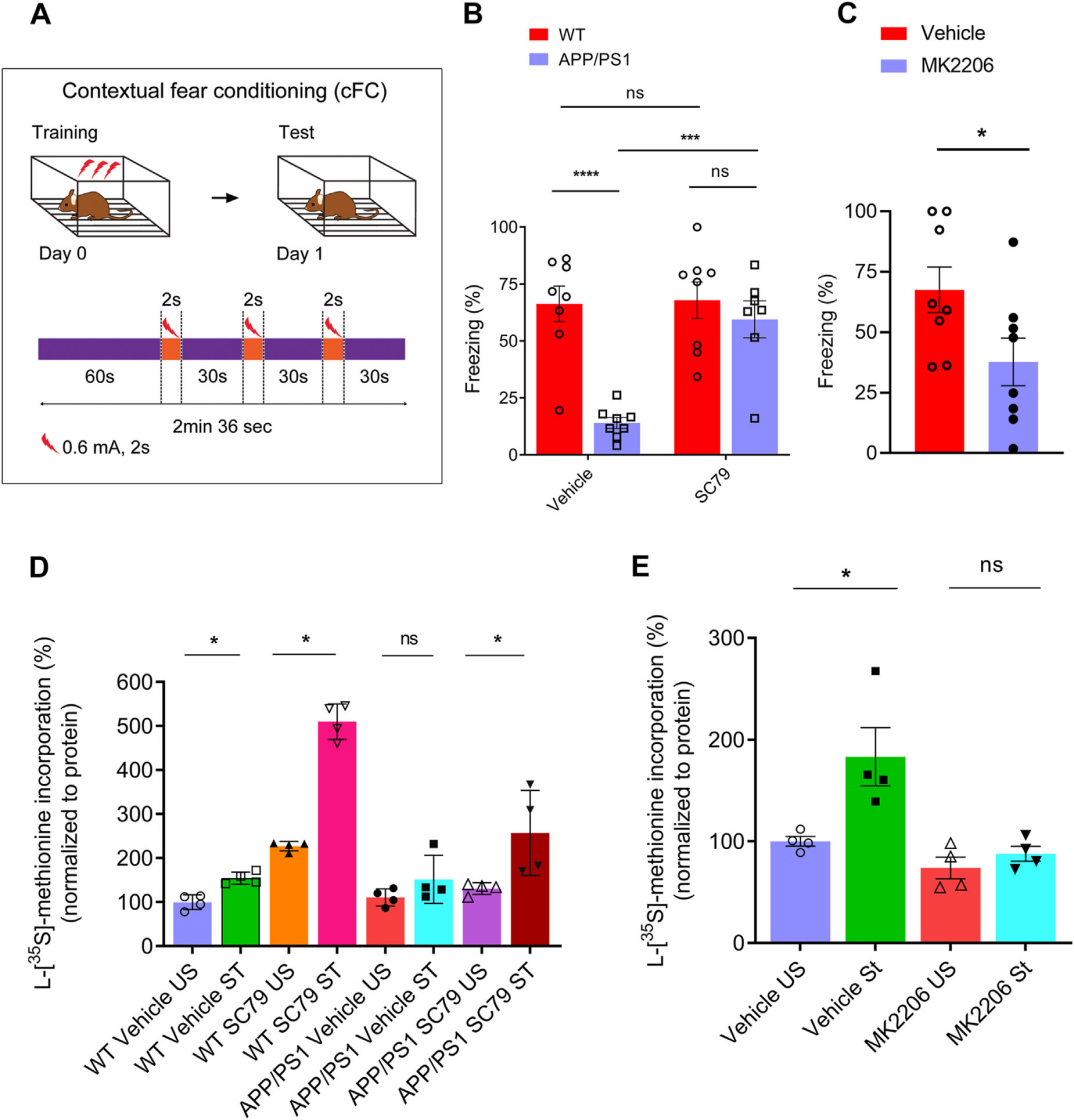
**Pharmacological activation and inhibition of Akt activity affects memory recall and activity-dependent synaptic protein synthesis in APP/PS1 male mice.***A*, schematic of the cFC behavioral design. *B*, WT and APP/PS1 mice were injected with vehicle or SC79 (Akt activator). Contextual fear conditioning memory (freezing response) was assessed 24 h after training. Representative *bar graph* showing the injection of SC 79 in APP/PS1 mice displayed more freezing behavior compared to age-matched, vehicle-treated APP/PS1 mice. Statistical significance indicated as ∗∗∗*p* < 0.001 and two-way ANOVA, followed by Tukey’s post test. Statistical analysis: interaction: F (1, 27) =9.992, *p = 0.*0039; SC79: F (1, 27) =11.50, *p* = 0.0022; shRNA: F (1, 27) =19.10, *p* = 0.0002). WT-vehicle *versus* APP/PS1-vehicle, *p* < 0.0001; WT-vehicle *versus* WT-SC79, *p* = 0.9984; WT-vehicle *versus* APP/PS1-SC79, *p* = 0.9032; APP/PS1-vehicle *versus* WT-SC79, *p* < 0.0001; APP/PS1-vehicle *versus* APP/PS1-SC79, *p* = 0.0006, 95% confidence interval [−72.81 to −18.16]; WT-SC79 *versus* APP/PS1-SC79, *p* = 0.8345. n = 8. *C*, C57BL/6 mice were injected with vehicle (DMSO) or MK2206 (Akt inhibitor). *Bar graph* showing the assessment of freezing response 24 h after training and freezing response was significantly decreased in C57BL/6 mice treated with MK2206 as compared to control mice injected with vehicle. Statistical analysis: vehicle *versus* MK2206, Mann–Whitney U test, *p* = 0.0368. n = 8 mice, values are presented as mean ± SEM. ∗ indicates *p* < 0.05. *D*, KCl stimulation of synaptoneurosomes of WT and APP/PS1 mice treated with SC79 showed significant increase in the incorporation of [^35^S]-L-methionine over unstimulated samples in APP/PS1 and WT mice. Statistical analysis: Mann–Whitney U test, WT-vehicle-US *versus* WT-vehicle-St, *p* = 0.0286, WT-SC79-US *versus* WT-SC79-St, *p* = 0.0286, APP/PS1-vehicle-US *versus* APP/PS1-vehicle-St, *p* = 0.200, APP/PS1-SC79-US *versus* APP/PS1-SC79-St, *p* = 0.0286. n = 4. Values are presented as mean ± SEM. ∗ indicates *p* < 0.05. *E*, KCl stimulation of synaptoneurosomes of C57BL/6 mice treated with MK2206 did not show any increase in the incorporation of [^35^S]-L-methionine compared to unstimulated samples. Statistical analysis: Mann–Whitney U test, vehicle-US *versus* vehicle-St, *p* = 0.0143, MK2206-US *versus* MK2206-SC79-St, *p* = 0.4857. n = 4. Values are presented as mean ± SEM. ∗ indicates *p* < 0.05. APP/PS1, amyloid precursor protein/presenilin-1; cFC, contextual fear conditioning; DMSO, dimethyl sulfoxide; MK, MK2206-injected mice; SC79-SC79-injected mice; St, stimulated; Vehicle, DMSO-injected mice; US,unstimulated.

To investigate the effect of Akt perturbation (through Akt activation or inhibition) on memory recall, we injected Akt activator (SC79) intrathecally in three-month-old WT and APP/PS1 mice after contextual fear memory training (Fig. 5*A*) and assessed recall 24 h after the training. Similarly, we injected Akt inhibitor (MK2206) intrathecally into 3-month-old C57BL/6 mice after training and assessed recall 24 h later. APP/PS1 mice had significantly impaired memory recall compared to that of WT mice (Fig. 5*B*, interaction: F (1, 27) = 9.992, *p* = 0.0039; SC79: F (1, 27) = 11.50, *p* = 0.0022; genotype: F (1, 27) = 19.10, *p* = 0.0002). However, APP/PS1 mice injected with Akt activator (SC79) displayed more freezing behavior (rescued deficits in memory recall) than those injected with vehicle (Fig. 5*B*). Further, we observed substantial less freezing behavior in C57BL/6 mice injected with Akt inhibitor (MK2206) than those injected with vehicle (Fig. 5*C*, *p* = 0.0368).

Further, we conducted experiments to study the role of Akt1 in activity-dependent protein synthesis in APP/PS1 and C57BL/6 mice. We found that stimulation of synaptoneurosomes with KCl isolated from WT mice treated with SC79 resulted in a significant increase in the incorporation of [^35^S]-L-methionine–an indicator for activity-dependent protein synthesis (Fig. 5*D*, WT-vehicle-US *versus* WT-vehicle-St, *p* = 0.0286, Hodges–Lehmann estimate: −54.30; WT-SC79-US *versus* WT-SC79-St, *p* = 0.0286, Hodges–Lehmann estimate: −294.2; APP/PS1-vehicle-US *versus* APP/PS1-vehicle-St, *p* = 0.200, APP/PS1-SC79-US *versus* APP/PS1-SC79-St, *p* = 0.0286, Hodges–Lehmann estimate: −119.6). The incorporation of [^35^S]-L-methionine by KCl stimulation was, however, unaffected in APP/PS1 mice at 3 months of age treated with vehicle (Fig. 5*D*). Intriguingly, we found that activation of Akt through a pharmacological agent SC79 and stimulation of synaptoneurosomes with KCl isolated from APP/PS1 mice induced activity-dependent incorporation of [^35^S]-L-methionine compared to that of unstimulated SC79-treated APP/PS1 mice (Fig. 5*D*). C57BL/6 mice in which Akt was inhibited with the pharmacological agent MK2206 had significantly less activity-dependent protein synthesis than that of vehicle-treated mice (Fig. 5*E*, Vehicle-US *versus* Vehicle-St, *p* = 0.0143; MK2206-US *versus* MK2206-St, *p* = 0.4827). Our results provide evidence that Akt activation ameliorates deficits in both memory recall and activity-dependent protein synthesis at the synapse in the hippocampus of APP/PS1 mice.

## Discussion

Our research uncovered novel functions for Akt1/mTOR signaling proteins in the hippocampus of an AD mouse model. We demonstrated for the first time that in presymptomatic ages in both post nuclear supernatant and synaptosomes of the male APP/PS1 hippocampus, the Akt1/mTOR signaling pathway proteins are significantly downregulated. In synaptoneurosomes of APP/PS1 mice of presymptomatic ages, activity-dependent protein synthesis is impaired. Akt1 is essential for BDNF-stimulated protein synthesis at the synapse but not in the soma, supporting the proposition that Akt1 is required for synaptic plasticity. Akt activation by SC79 improves deficits in both memory recall and activity-dependent protein synthesis in the hippocampus of APP/PS1 mice. Akt inhibitor impairs memory recall and activity-dependent protein synthesis in the hippocampus of C57BL/6 mice. This is the first study to show, in a mouse model of AD, global downregulation of the Akt1/mTOR pathway in the hippocampus and the role of Akt1 in activity-dependent synaptic protein synthesis. However, our study primarily focused on one of the AD mouse models and utilized mostly one age group (adolescents, at 1 month) and in some experiments, young adults, at 3 months. Due to these limitations, these findings warrant further examination using other mouse models of AD with different age groups.

Sex is well established as one of the risk factors for AD pathology, with several studies demonstrating sex differences in AD (53, 54, 55, 56, 57, 58, 59). According to a previous report, male APP/PS1 mice showed memory recall deficits earlier than female APP/PS1 mice (4, 58). We found the early dysregulation of Akt/mTOR signaling pathways in the synaptosomes isolated from the brain cortex of APP/PS1 mice (43). We therefore sought to elucidate the role of A-β in hippocampus dependent long-term memory and Akt/mTOR signaling pathways in male mice well before the manifestation of clinical symptoms. Female mice are demonstrated to exhibit greater resilience to early-stage pathological changes when compared to males (58, 60, 61). Because of sex hormones like estrogen, female mice, as long as their estrogen levels remain intact, are better protected than male mice (62, 63). However, as soon as their estrogen levels begin to decline, the cognitive deficits and other AD pathologies manifest rapidly (61, 62, 64, 65, 66, 67). Therefore, anticipating that there may be variations in the observed results based on sex, we do not compare male and female mice in this study. Our future research will further investigate whether there is sex-specific dysregulation of Akt/mTOR signaling in the hippocampus.

In AD, Aβ oligomers–pathogenic molecules that negatively regulate many signaling pathways necessary for synaptic function and plasticity–trigger dendritic spine loss, synaptic dysfunction, impaired memory formation, and neuronal degeneration (68). Dysregulation of the Akt/mTOR signaling pathway has been associated with AD pathology in both *in vivo* animal models of AD and in postmortem brain tissues from AD patients (21, 42, 69, 70, 71). In both healthy and pathological situations, the PI3K/Akt/mTOR signaling pathways are critical for cell survival, proliferation, synaptic function, and memory formation. Knock out of isoform-specific Akt1 failed to increase late-phase LTP–induced protein synthesis in hippocampal slices (20). Despite its role in learning and memory, the Akt1/mTOR signaling cascade has not been studied in the hippocampus of an AD mouse model at an early age.

Therefore, we assessed the phosphorylation of Akt1 and mTOR signaling pathways in post nuclear supernatant and synaptosomes isolated from the hippocampus of WT and APP/PS1 mice at 1 month. We found that phosphorylation of Akt1 and GSK3β was diminished in both post nuclear supernatant (Fig. 1, *A*–*C*) and synaptosomes (Fig. 1, *D*–*F*) of the hippocampus of APP/PS1 mice, while we did not observe this downregulation in post nuclear supernatant of the brain cortex in one-month-old APP/PS1 mice (Fig. S1, *A*–*C*). Our results clearly demonstrate that disruption of the Akt1 signaling cascade appears earlier in the hippocampus than in the cerebral cortex of APP/PS1 male mice.

In the hippocampus of one-month-old APP/PS1 male mice, we observed dysregulation of phosphorylation of the mTOR signaling pathway and its downstream targets, S6 ribosomal protein and 4E-BP1, in both post nuclear supernatant (Fig. 2, *A*–*C*) and synaptosomes (Fig. 2, *D*–*F*). Post nuclear supernatant from the brain cortex of APP/PS1 mice aged 1 month demonstrated no dysfunction in the mTOR signaling pathway (Fig. S2, *A*–*C*). Our novel findings revealed for the first time the temporal difference in Akt1/mTOR dysregulation between the hippocampus and brain cortex of 1-month-old APP/PS1 mice. Alteration in the mTOR signaling events also appears earlier in the hippocampus than in the brain cortex. Our results from synaptosomes isolated from the hippocampus of one-month-old APP/PS1 mice were identical to those previously reported in the cerebral cortex (43), but differed from those of post nuclear supernatant from the cerebral cortex. Increased levels or activation of Akt1 have been demonstrated to underlie the neurotrophin-, estrogen- (72), and lipoic acid-mediated (73) neuroprotection in AD model systems. Our findings suggest that dysregulation of Akt1/mTOR signaling cascades in the hippocampus could contribute to the impairment of hippocampal dependent long-term memory, synaptic, and other cognitive functions.

General protein synthesis disruption impairs long-term memory and synaptic plasticity (74, 75, 76). Local protein synthesis at synapses, both basal and activity-dependent, affects synaptic function, plasticity, and cognition (43, 77, 78, 79). Because activity-dependent synaptic protein synthesis is a highly reliable indicator of synaptic function and plasticity (77, 78, 79), early dysfunction of activity-dependent local protein synthesis in the pathogenesis of AD would have far-reaching consequences for synapse maintenance and plasticity, culminating in memory and cognitive deficits (80, 81, 82). We previously found activity-dependent protein synthesis to be impaired both in synaptoneurosomes prepared from human brain cortex of AD subjects and in brain cortex from 1-month-old APP/PS1 mice (43). Compared to age-matched WT mice, this deficiency was sustained even at middle age in APP/PS1 mice. However, the dysfunction of activity-dependent protein synthesis in the hippocampus in APP/PS1 male mice remains poorly studied. Hence, we assessed basal and activity-dependent protein synthesis in post nuclear supernatant and synaptoneurosomes from the hippocampus of WT and APP/PS1 mice at one and 3 months. KCl stimulated activity-dependent protein synthesis was not affected in post nuclear supernatant of APP/PS1 mice at 1 month (Fig. 3*A*) and 3 months. (Fig. 3*C*). However, we failed to observe activity-dependent synaptic protein synthesis in the hippocampus of APP/PS1 mice at 1 month (Fig. 3*B*) and 3 months (Fig. 3*D*). These findings support our prior findings that activity-dependent protein synthesis is reduced in synaptoneurosomes isolated from the cerebral cortex of APP/PS1 mice at different ages (43).

Akt/mTOR signaling pathway dysregulation has been studied in neurological disorders such as AD, traumatic brain injury, brain tumors, epilepsy, autism, diabetes, and aging process (21, 28, 42, 43). Akt1/mTOR signaling is demonstrably critical for activity-dependent dendritic protein synthesis (27, 79, 83, 84, 85). While Akt1 is involved in aberrant synaptic plasticity (75), in the AD mouse model, the role of Akt1 and mTOR signaling pathways in regulating baseline and activity-dependent synaptic protein synthesis in the hippocampus has not been studied. Hence, to study the role of Akt1 in activity-dependent protein synthesis, we downregulated Akt1 with shRNA in primary hippocampal neurons. While BDNF-stimulated protein synthesis in the cell body (soma) of hippocampal neurons was unaffected by Akt1 knockdown (Fig. 4, *A* and *B*), BDNF-stimulated synaptic protein synthesis was significantly decreased by the downregulation of Akt1 in hippocampal neurites (Fig. 4, *C* and *D*). We therefore suggest that the Akt1 or the Akt1/mTOR signaling pathway may be a potential mechanistic target for driving activity-dependent protein synthesis in neurites. Our results clearly demonstrate the essential of Akt1 for hippocampal activity-dependent synaptic protein synthesis and synaptic plasticity.

Akt activation has been linked to learning and memory (86, 87, 88, 89, 90). We therefore evaluated the efficacy of Akt activation in recovering deficits in memory recall in an AD mouse model. Activation of Akt by SC79 rescued deficits in both memory recall (Fig. 5*B*) and KCl stimulated synaptic protein synthesis in male APP/PS1 mice at 3 months (Fig. 5*D*). In contrast, inhibition of Akt by MK2206 impaired memory recall and KCl stimulated synaptic protein synthesis in the hippocampus of male C57BL/6 mice at 3 months (Fig. 5, *C* and *E*). Our results indicate that Akt1 acts as a restraint on mTOR-regulated activity-dependent protein synthesis and synaptic plasticity (20, 91, 92, 93).

Akt1, which has been implicated in other brain disorders and contributed to the impairment of synaptic plasticity (94), is a well-established effector for neuregulin1 (NRG1–ERBB4–PI3K–AKT1 signaling cascade) pathway, and NRG1, *via* the ErbB4 receptor, prevented the Aβ_1-42_–induced impairment of LTP in hippocampal slices (95). In a transgenic mouse model of AD, NRG1 improved cognitive function (96, 97), and in the hippocampus of AD brains, NRG1 has been shown to be decreased (98). These reports may suggest decreased protein synthesis, possibly as a result of Akt1 dysregulation; our findings demonstrated that Akt1/mTOR signaling cascade proteins were dysregulated early in the hippocampus but were not specific to synaptosomes as observed in the brain cortex of APP/PS1 mice (43). The decrease of activity-dependent protein synthesis in the hippocampus of APP/PS1 mice supports the connection between the dysregulation of the Akt1/mTOR signaling cascade and impaired synaptic and cognitive functions in AD. Depletion of Akt1 by shRNA resulted in impairment of BDNF-stimulated protein synthesis in the hippocampal neurons. Further, SC79 rescued both KCl stimulated synaptic protein synthesis and memory recall deficits in APP/PS1 mice during presymptomatic ages, while MK2206 augmented the memory recall deficits and abolished KCl stimulated synaptic protein synthesis in C57BL/6 mice. Overall, our study demonstrated the importance of the Akt1/mTOR signaling cascade as a critical pathway for maintaining synaptic strength, activity-dependent protein synthesis, long-term memory, synaptic plasticity, and other higher order cognitive functions in AD during presymptomatic ages.

## Experimental procedures

### Chemicals and reagents

Sucrose (Cat. No. 84097), N,N,N′,N′-tetramethylethylenediamine (Cat No. T7024), DL-DTT (Cat. No. D9779), 2-amino-2-(hydroxymethyl)-1,3-propanediol (Cat. No. T1503), 4-(2-hydroxyethyl) piperazine-1-ethanesulfonic acid (Cat. No. 54457), DNase I (Cat. No. D-4513), papain (Cat. No. P-4762), and poly-D-lysine hydrobromide (Cat. No. P6407), β-glycerophosphate disodium salt hydrate (Cat. No. G9422), sodium orthovanadate (Cat. No. S6508), sodium fluoride (Cat. No. 201154), puromycin (Cat. No. P8833), and other biochemical’s were purchased from Sigma-Aldrich. SC79 (Cat. No. 4635) was purchased from Tocris Bioscience, MK-2206 (Cat. No. S1078) was purchased from Selleck Chemicals LLC. S^35^-L-methionine was procured from Board of Radiation and Isotope Technology. Aprotinin (Cat. No. 10236624001), leupeptin (Cat. No. 11017101001), antipain dihydrochloride (Cat. No. 11004646001), pepstatin (Cat. No. 11359053001), PMSF (Cat. No.10837091001) were purchased from F. Hoffmann-La Roche Ltd. ProLong gold antifade mountant with 4',6-diamidino-2-phenylindole (Cat. No. P36931), GlutaMAX supplement (Cat. No. 35050061), penicillin-streptomycin (Cat. No. 15140122), neurobasal-A medium (Cat. No. 10888022), B-27 Supplement (50×), serum free (Cat. No. 175040444), and Bicinchoninic acid protein assay kits were purchased from Thermo Fisher Scientific Inc. Precision plus protein dual color standards (Cat. No. 1610374), hydrophilic polyvinylidene difluoride membrane for Western blotting (Cat. No. 1620177), clarity, and clarity max enhanced chemiluminescence Western blotting substrates (Cat. No. 1705061 and Cat. No. 1705062) were purchased from Bio-Rad Laboratories, Inc.

### Antibodies

Anti-puromycin antibody (Cat. No. MABE343) from Millipore and anti-β-tubulin mouse mAb (Cat. No. T8328) was purchased from Sigma-Aldrich Chemical Company (Cat. No. T4026, RRID: AB_477577). Anti-MAP2 antibody (Cat. No. ab183830, RRID:AB_2895301) was purchased from Abcam. Rabbit anti-Akt1 (Cat. No. 2938, RRID: AB_915788), phospho-Akt (Thr308) (D25E6) (Cat. No.13038, RRID: AB_262944), Phospho-Akt1 (Ser473) (Cat. No. 4060, RRID: AB_2315049), phospho-GSK-3β (Ser9) (Cat. No. 9336, RRID: AB_331405), anti-GSK-3β (3D10) (Cat. No. 9832, RRID: AB_10839406), anti-mTOR (L27D4) (Cat. No. 4517, RRID: AB_1904056), phospho-mTOR (Ser2448) (D9C2) (Cat. No. 5536, RRID: AB_10691552), anti-4E-BP1, phospho (Thr37/Thr46) (Cat. No. 2855, RRID: AB_560835), anti-4E-BP1 (53H11) (Cat. No. 9644, RRID: AB_2097841), phospho-S6 ribosomal protein (Ser240/244) antibody (Cat. No. 2215, RRID: AB_331682), S6 ribosomal protein (54D2) mouse mAb (Cat. No. 2317, RRID: AB_2238583) were purchased from Cell Signaling Technology, Inc. Secondary antibodies, (Horse anti-mouse IgG antibody (H + L), peroxidase (Cat. No. PI-2000), goat anti-rabbit IgG antibody (H + L), peroxidase (Cat. No. PI-1000)) conjugated to peroxidase were purchased from Vector Laboratories, Inc.

### Animals

The APP_Swe_/PS1ΔE9 (APP/PS1) double transgenic mice on C57BL/6J background were purchased from the Jackson Laboratory (https://www.jax.org/strain/005864; RRID: MMRRC_ Stock_ No: 034832-JAX) (99). WT and APP/PS1 mice were bred and maintained at the Institutional Central Animal Facility. All mice were housed in a temperature-controlled room on a 12 h light/12 h dark cycle and these rooms were maintained under sterile and pathogen-free conditions. All mice had *ad libitum* access to food and water. All the animal experiments were conducted in compliance with the ARRIVE guidelines. All experimental protocols and procedures were approved by the Institutional Animal Ethics Committee and are in accordance with the guide for the care and use of laboratory animals. We confirmed the amyloid pathology by thioflavin-S and amytracker staining of the brain sections from 4-month-old APP/PS1 mice because A-β aggregates appears in cortical areas at this age (100) (Fig. S3).

### Isolation of post nuclear supernatant and synaptosomes

WT and APP/PS1 mice were sacrificed under anesthesia by intramuscular injection of ketamine (80–100 mg/kg) and xylazine (5–10 mg/kg), followed by a secondary method of euthanasia such as cervical dislocation and decapitation. Mouse brain was removed quickly and brain regions such as hippocampus and cortex were dissected out using standard anatomical landmarks. The hippocampus tissue was flash-frozen in liquid nitrogen and stored at −70 °C immediately until use. The experimental procedure for isolation of post nuclear supernatant and synaptosomes were followed as mentioned earlier (43). Briefly, mouse hippocampus tissue, pooled from six animals for one experimental number (n = 1), was thawed on ice prior to use and minced in ice-cold homogenization buffer (5 mM Hepes buffer, pH 7.4, containing 0.32 M sucrose, 50 mM sodium fluoride, 1 mM sodium orthovanadate, 2 μg/ml aprotinin, 10 μg/ml leupeptin, 7 μg/ml pepstatin A, 100 μg/ml of PMSF, and 50 μg/ml anti-pain). After mincing, the hippocampus tissue was transferred to a precooled *Potter*-Elvehjem homogenizer and homogenized in ten volumes of homogenization buffer using electronic motor-controlled homogenizer (Glas-Col). The tissue homogenate was transferred to precooled micro centrifuge tubes and spun down at 1000*g* for 10 min at 4 °C. The post nuclear supernatant was collected from each tube and transferred to new precooled micro centrifuge tubes. The post nuclear supernatant was centrifuged at 13,000*g* for 15 min at 4 °C. After centrifugation, the pellet was resuspended in 5 mM Tris (pH 8.1) containing 0.32 M sucrose buffer supplemented with protease and phosphatase inhibitors. Thereafter, we prepared the three discontinuous sucrose gradient tubes (0.85–1–1.2 M) and the resuspended crude mitochondrial suspension was layered on top of the 0.85 M sucrose gradient and centrifuged at 85,000*g* for 2 h at 4 °C using table top ultracentrifuge (Optima MAX-XP ultracentrifuge-Beckman-Coulter). Synaptosomal fraction obtained at the interface of 1 M, and 1.2 M sucrose gradient was collected and washed twice with 5 mM Tris (pH 8.1) buffer and resuspended in minimum amount of homogenization buffer, aliquoted, snap frozen in liquid nitrogen and stored in −70 °C. Protein concentration was measured by bicinchoninic acid protein assay.

### SDS-PAGE and immunoblotting

The post nuclear supernatant and synaptosomal fractions were subjected to SDS-PAGE. Proteins were transferred from the gel to PVDF membrane by electroblotting (101). Membranes were blocked in tris-buffered saline (TBS; 10 mM Tris, pH 8, 150 mM NaCl) supplemented with 5% (w/v) bovine serum albumin for 1 h at room temperature and immunoblotted with respective primary antibodies and incubated at 4 °C overnight. The following day, all immunoblots were washed with TBS with tween (10 mM Tris, pH 8, 150 mM NaCl, 0.05% Tween 20) and incubated for 1 h at room temperature in respective secondary antibodies in TBS supplemented with 5% (w/v) dried skimmed milk powder. Thereafter, immunoblots were washed with TBS with tween. Immunoreactive bands were detected by enhanced chemiluminescence (Clarity Western ECL blotting substrate, Bio-Rad). Immunoreactive signals were acquired using the Bio-Rad Chemidoc-XRS and analyzed with Imagelab software (Bio-Rad) (https://www.bio-rad.com/ImageLab).

### Isolation of synaptoneurosomes and L-[^35^S]-methionine incorporation assay

Synaptoneurosomes were isolated from mouse hippocampus tissue and L-[^35^S]-methionine incorporation assay was carried out as reported earlier (43). Hippocampus tissue, pooled from two animals for one experimental number (n = 1), was homogenized with ten volumes of translation buffer (118 mM NaCl, 4.7 mM KCl, 1.2 mM MgSO_4_, 2.5 mM CaCl_2_, 1.53 mM KH_2_PO_4_, 212.7 mM glucose, and 1 mM DTT) containing 200 μg/ml chloramphenicol, 30 U/ml RNAse inhibitor, protease, and phosphatase inhibitors. The homogenate was then passed sequentially through two 100 μm and one 10 μm nylon mesh filters (Millipore). Thereafter, the filtered homogenate was centrifuged at 1500*g* at 4 °C for 10 min. After centrifugation, supernatant and the pellet were obtained. Pellet, containing synaptoneurosomes, was resuspended in translation buffer. Synaptoneurosomes were stimulated with 50 mM KCl at 37 °C for 15 min in presence of 50 μCi L-[^35^S]-methionine. Unstimulated synaptoneurosomes were incubated at 37 °C for 15 min in the presence 50 μCi L-[^35^S]-methionine alone. After incubation, proteins were precipitated from synaptoneurosomes using equal volume of ice cold 10% (w/v) trichloroacetic acid. Precipitated protein pellet was washed extensively with ice cold 5% (w/v) trichloroacetic acid, followed by washes with ice-cold methanol until the washes showed no detectable radioactivity. The washed protein pellets were resuspended in 0.1 N sodium hydroxide and radioactivity was measured using liquid scintillation counter.

### Preparation of primary hippocampal neuronal culture

Primary hippocampal pyramidal neurons were prepared from postnatal day 0 to 1 (P0-P1) C57BL/6 mouse brain hippocampus (102). After removing meninges and cortical tissue, hippocampus was dissected out. Hippocampal neurons were dissociated using mechanical trituration in 0.1% DNase and 0.25% papain solution containing HHGN (1 × Hanks' Balanced Salt Solution, 12.5 mM Hepes-buffered sterile saline, D-glucose). Hippocampal neurons were seeded on cover slips precoated with poly-D-lysine (0.1 mg/ml) in 12-well plates. Neurobasal medium supplemented with 0.5 × B27, 2 mM L-GlutaMAX, and 100 μg/ml penicillin/streptomycin was used to grow the cells in serum-free medium conditions and maintained at 37 °C in 5% CO_2_ for 3 weeks.

### Downregulation of Akt1 in Neuro2a cells and primary hippocampal neurons

The shRNA oligonucleotides against sc-Akt1 (scrambled control of Akt1) and sh-Akt1 (shRNA against Akt1) were synthesized, and the sequences are shRNA-Akt1F-5′-TTTGTCAAACTCGTTCATGGTCAAAGTTCTCTTGACCATGAACGAGTTTGATTTTT-3′, shRNA-Akt1R-5′-CTAGAAAAATCAAACTCGTTCATGGTCAAGAGAACTTTGACCATGAACGAGTTTGA-3′, scRNA-Akt1F-5′-TTTGCTCGACTTCGTATTAAGACAAGTTCTCTGTCTTAATACGAAGTCGAGTTTTT-3′, scRNA-Akt1R-5′-CTAGAAAAACTCGACTTCGTATTAAGACAGAGAACTTGTCTTAATACGAAGTCGAG-3′. The pMD2.G and psPAX2 were a gift from Dr Didier Trono (Addgene Plasmid# 12259, 12260). The pRRLsinPPTeGFP plasmid (pLenti-GFP) was kindly provided by Dr Philip A. Barker (McGill University). scRNA-Akt1 and shRNA-Akt1 were subcloned into a pRRLsinPPTeGFP vector to generate pRRLsinPPTscRNA-Akt1 and pRRLsinPPTshRNA-Akt1, respectively. Lentiviral particles were produced using second generation lentiviral systems in HEK293T cells; particles were purified by ultracentrifugation and resuspended in Neurobasal-A medium, and the amount of active particles was determined by titration in HEK293T cells. Neuro2a cells were transduced with lenti-scRNA-Akt1 and lenti-shRNA-Akt1. After 3 days of transduction, cells were washed with ice cold 1×PBS buffer and lysed in 2× Laemmli buffer. The cell lysates were resolved on SDS-PAGE, followed by immunoblotting with an antibody against Akt1 and subsequently, the PVDF membrane was stripped and reprobed for β-tubulin as a loading control. Primary hippocampal neurons from C57BL/6 mice were transduced at *DIV18* with scRNA-Akt1 (scrambled control of Akt1) and shRNA-Akt1 (shRNA against Akt1) lentiviral particles to determine the effects of Akt1 downregulation on BDNF-stimulated protein synthesis.

### Protein synthesis assay in primary hippocampal neurons

Protein synthesis in primary neurons was assayed as described elsewhere (103). Primary hippocampal neurons (*DIV21*) were stimulated with 50 ng/ml of BDNF for 1 h at 37 °C, followed by treatment with 3 μM puromycin for 7 min at 37 °C. After brief washing, treatment was terminated by fixation with 4% (w/v) paraformaldehyde and 4% (w/v) sucrose for 20 min at room temperature. This was followed by permeabilization with 0.3% (v/v) Triton X-100. Cells were then coimmunostained with antibodies against MAP2 and puromycin for 1 h at room temperature.

### Confocal image acquisition and analysis

Confocal images were acquired using Carl Zeiss LSM780 laser scanning system using Argon 488 laser for puromycin. Helium-neon 594 laser was used to visualize MAP2. Oil immersion objective 63×/1.40 was used and *z*-stack images were captured. All confocal images for puromycin and MAP2-labeled neuronal soma and neurites were acquired under identical conditions and analyzed after blinding. Quantification of puromycin levels measured as puromycin intensity was performed using MetaMorph software (MetaMorph, version 7.8.0.0, 2013, Molecular Devices, LLC) (http://www.moleculardevices.com/Products/Software/Meta-Imaging-Series/MetaMorph.html). Confocal *z*-stack images were loaded onto MetaMorph software and maximum intensity projection was generated. After background subtraction and thresholding, mask was generated around the soma and dendrite of interest (including the spines), and puromycin intensity was measured.

### Administration of Akt pharmacological modulators

SC-79 (Akt activator; 1 mg/ml) or MK-2206 (Akt inhibitor; 1 mg/ml) were freshly dissolved in dimethyl sulfoxide (3% in normal saline) and subsequently injected intrathecally at a dose of 0.05 mg/mouse (50 μl) or dimethyl sulfoxide in 3% in normal saline (50 μl) as a vehicle using three-months-old male WT and APP/PS1 mice.

### Contextual fear conditioning

All experiments were carried out with 3 months old male mice. Fear conditioning is a learning task where an organism forms a relation between a neutral stimuli/environment (context) with an aversive stimulus. The task involves two parts, the first is the training and the second is the recall step. Contextual fear conditioning, training context was rectangular in shape. Identity of the context was maintained with the presence of distinct odor (2% acetic acid (vol/vol)). The conditioning chamber was cleaned with 70% ethanol before and after each session. Mice were single housed for 10 days prior to commencement of training and were first handled for 5 min for 3 days. On training day mice were allowed to explore the training context for 1 min, and then received three-foot shocks, (2 s and 0.6 mA each, intertrial interval: 30 s). We assessed contextual fear memory by returning mice to the training context 24 h after fear conditioning without being subjected to a foot shock and analyzing freezing during a test period of 2 min. Their freezing response is recorded as a measure for memory. Freezing behavior is a natural rodent behavior in response to any aversive stimuli. In experiments, mice treated with pharmacological agents to inhibit or activate Akt protein were subjected to contextual fear conditioning prior to injections, along with appropriate controls.

### Statistical analysis

GraphPad Prism software (Prism 9.01, GraphPad Software Inc) (https://www.graphstats.net/graphpad-prism) was utilized for statistical analysis. For experiments with less than eight sample sizes in each group, nonparametric tests were used; and no normality checks were carried out. Continuous variables were first checked for normality using Shapiro-Wilk test and then *t* test or Mann–Whitney U test was used for statistical comparisons between two groups as appropriate. Statistical comparisons between WT and APP/PS1 groups treated without or with KCl were analyzed with the two-tailed, unpaired, Mann–Whitney “U” test. Two-way ANOVA, Tukey’s multiple comparisons test was carried out between sc-shRNA-Akt1 and shRNA-Akt1 groups for US (vehicle) or stimulated (with BDNF) conditions. The interaction effect between the two factors, that is, type of animals (WT and APP/PS1) and study groups (vehicle and SC79) were checked using two-way ANOVA.

## Data availability

The data that support the findings of this study are available upon reasonable request from the corresponding author.

## Supporting information

This article contains supporting information.

## Conflict of interest

The authors declare that they have no conflicts of interest with the contents of this article.
