# Supplementary figures and images for: Akt activation ameliorates deficits in hippocampal-dependent memory and activity-dependent synaptic protein synthesis in an Alzheimer’s disease mouse model
Source: J Biol Chem. 2024 Jan 3;300(2):105619. doi: 10.1016/j.jbc.2023.105619 (PMC10839450; doi:10.1016/j.jbc.2023.105619)

# Figure. S1

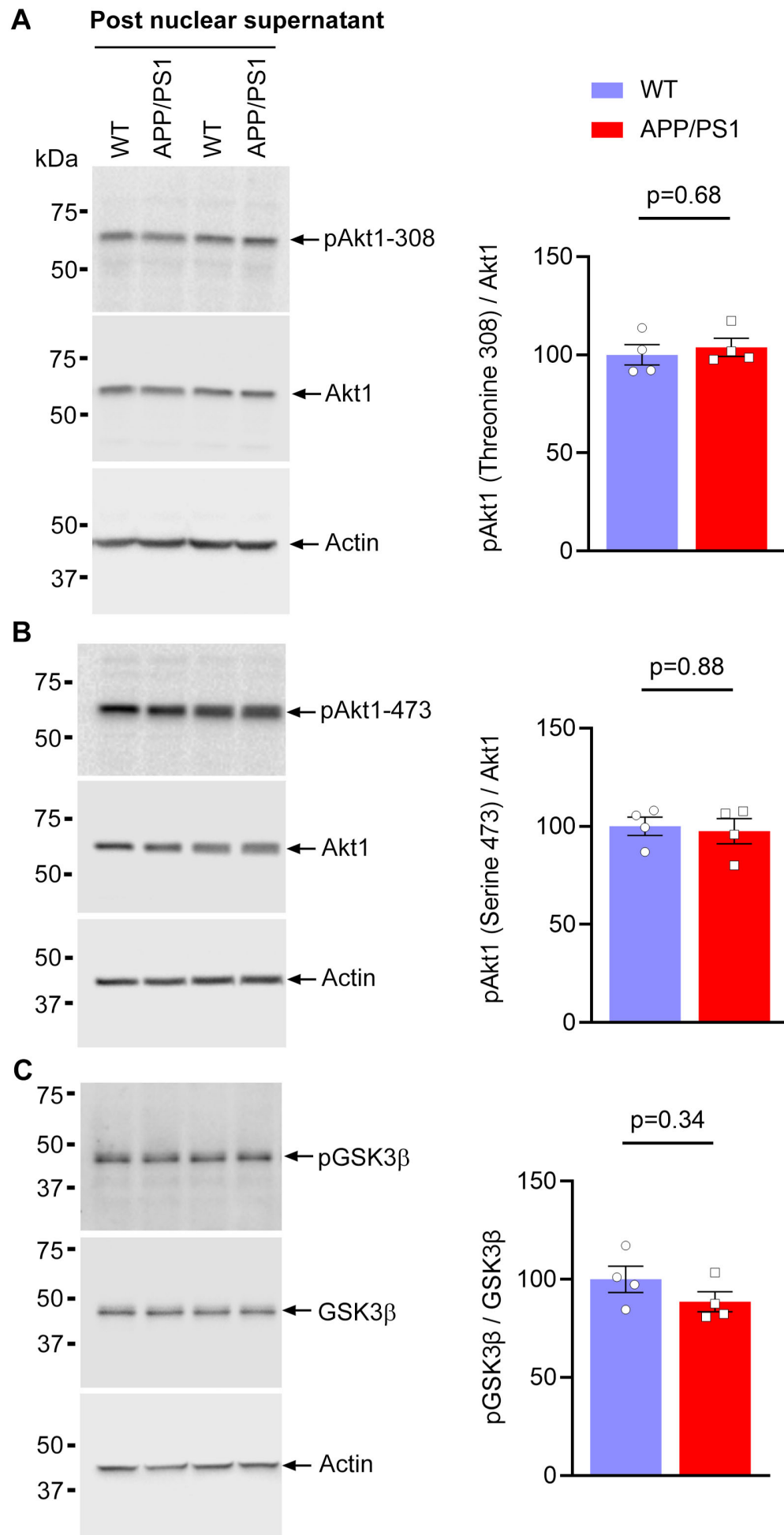

Supplement: Figure S1 [file mmc1.pdf]

**Figure. S2**

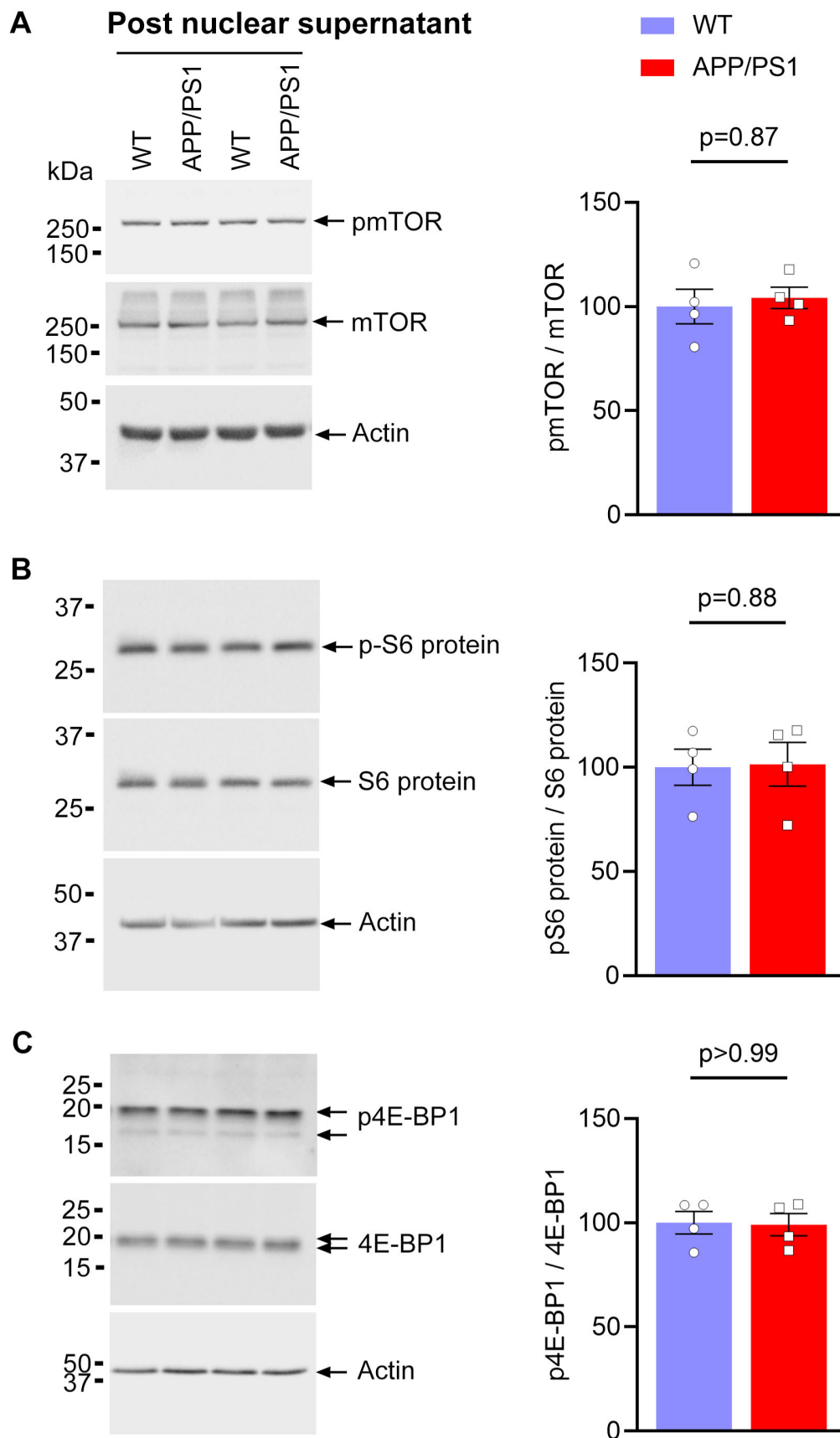

Supplement: Figure S2 [file mmc2.pdf]

Figure. S3

Male - 4 Months

Thioflavin-S

Amytracker 680

Overlay

Wild Type

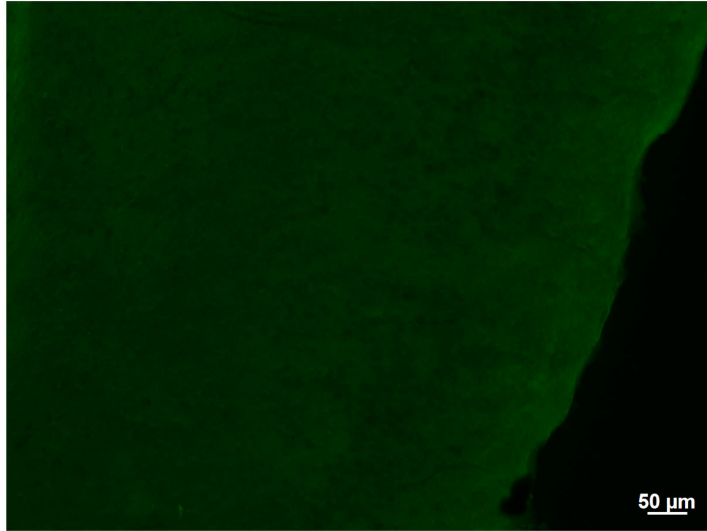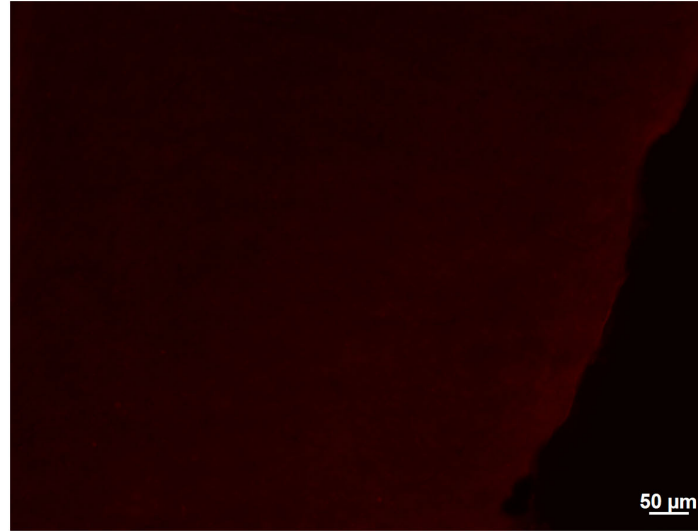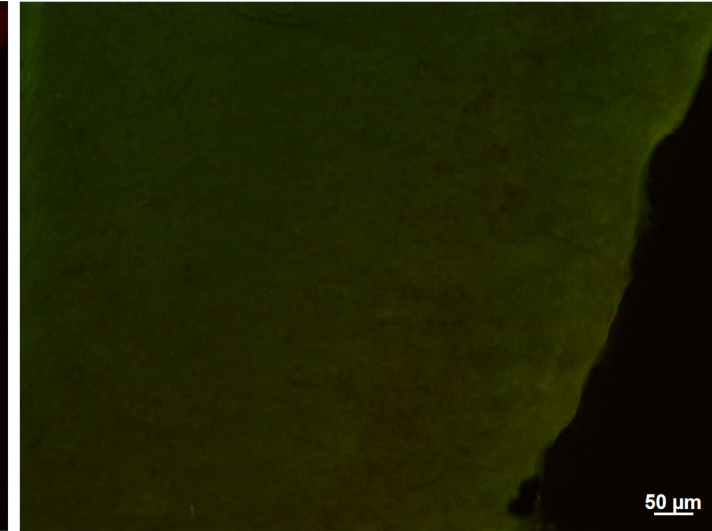

APP/PS1

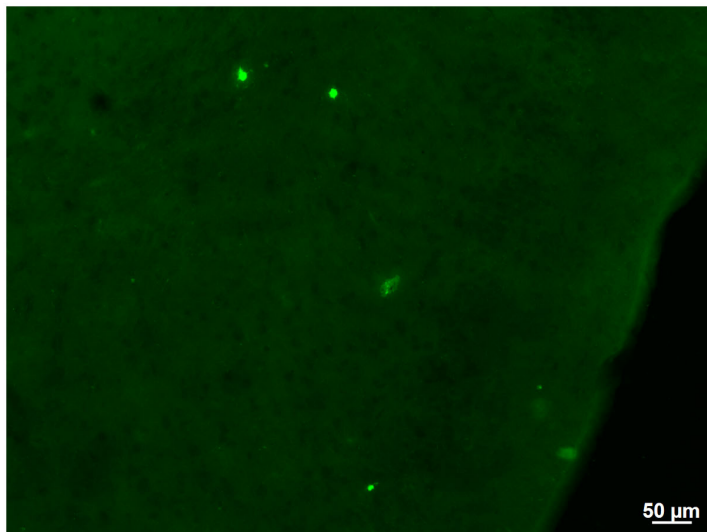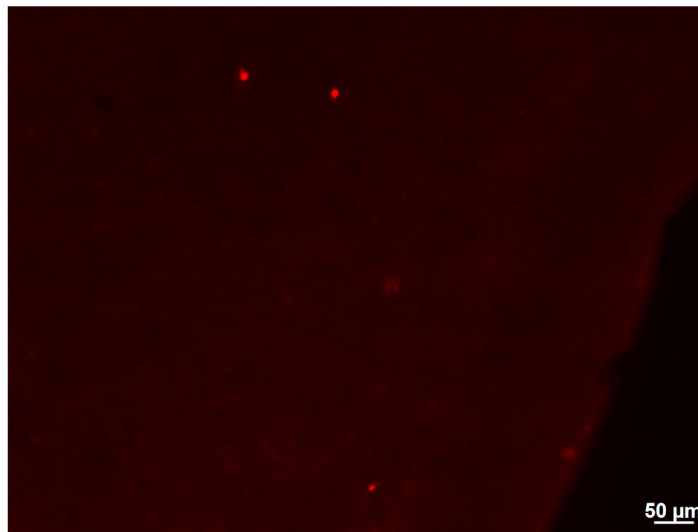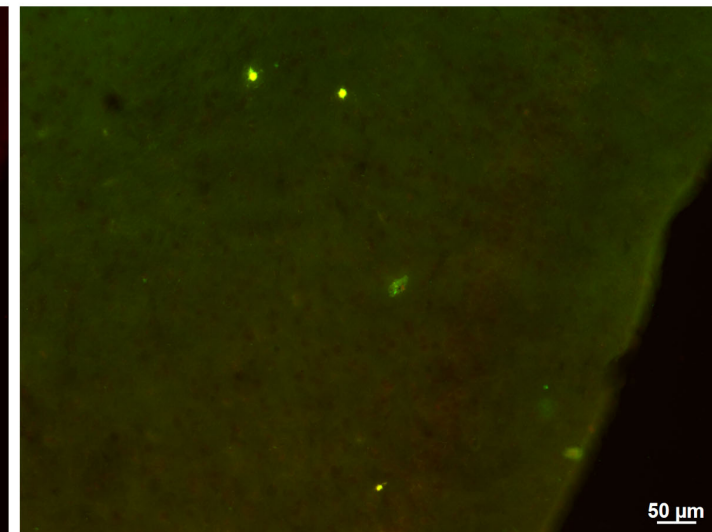

Supplement: Figure S3 [file mmc3.pdf]

**Figure. S4**

**A**

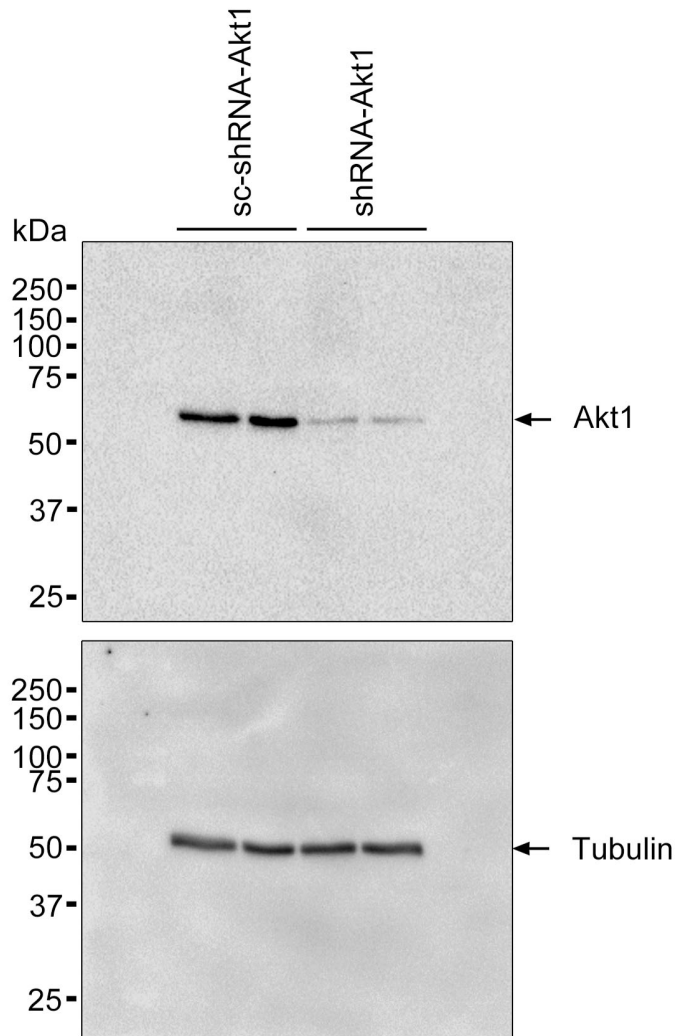

**B**

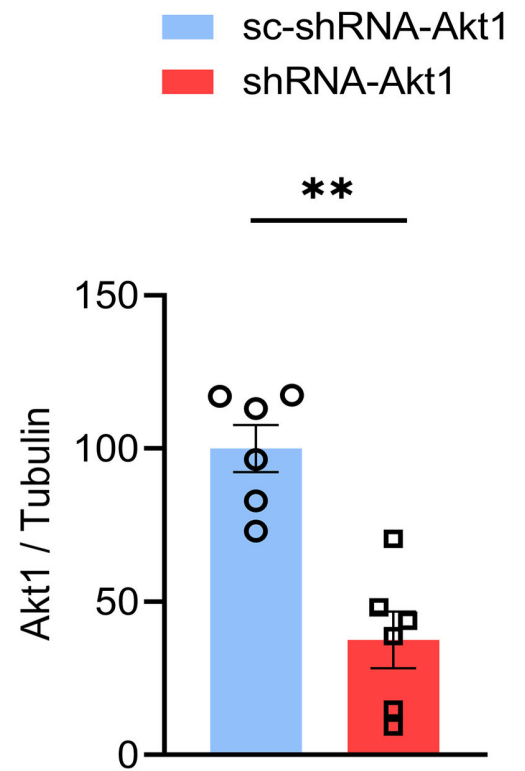

Supplement: Figure S4 [file mmc4.pdf]
