# Supplementary material for: Akt activation ameliorates deficits in hippocampal-dependent memory and activity-dependent synaptic protein synthesis in an Alzheimer’s disease mouse model
Source: J Biol Chem. 2024 Jan 3;300(2):105619. doi: 10.1016/j.jbc.2023.105619 (PMC10839450; doi:10.1016/j.jbc.2023.105619)
Supplement: Supporting Information [file mmc5.docx]

**Akt activation ameliorates deficits in hippocampal-dependent memory and activity-dependent synaptic protein synthesis in an Alzheimer’s disease mouse model**

Reddy Peera Kommaddi^1 # *^, Ruturaj Gowaikar^2#^, Haseena PA^1, 3^, Latha Diwakar^1^, Kunal Singh^2^, Amrita Mondal^1^

**Figure Legends To Supporting information**

**Figure S1.**

**Akt1 and GSK3β phosphorylation is not altered in post nuclear supernatant of brain cortex from one-month-old APP/PS1 male mice**

Representative immunoblots of (A) phosphorylation of Akt1 (threonine-308) and total Akt1 (p = 0.68) (n=4), (B) phosphorylation of Akt1 (serine-473) and total Akt1 (p = 0.88) (n=4), (C) phosphorylation of GSK3β and total GSK3β (p = 0.34) (n=4) in post nuclear supernatant from brain cortex of one-month-old (adolescent) WT and APP/PS1 mice. All immunoblots were stripped and reprobed for β-actin. Densitometric scan analysis depicted as bar graphs (right panels). Statistical analysis: Unpaired, two-tailed Mann-Whitney *U* test; Data are expressed as mean ±SEM. ‘p’ values are not significantly different from WT controls.

**Figure S2.**

**Mechanistic target of rapamycin (mTOR) signaling cascade proteins are not altered in post nuclear supernatant of brain cortex from one-month-old APP/PS1 male mice**

Western blots of the mTOR signaling components (A) phosphorylation of mTOR and total mTOR (p = 0.87) (n=4), (B) phosphorylation of S6 ribosomal protein and total S6 ribosomal protein (p = 0.88) (n=4), (C) phosphorylation of 4E-BP1 and total 4E-BP1 (p > 0.99) (n=4) in post nuclear supernatant (PNS) from brain cortex of one-month-old (adolescent) WT and APP/PS1 mice. All immunoblots were stripped and reprobed for β-actin. Quantification of protein band intensities is shown as bar graphs (right panels). Statistical analysis: Unpaired, two-tailed Mann-Whitney *U* test; Data are depicted as mean ±SEM. ‘p’ values are not significantly different from WT controls.

**Figure S3.**

**Thioflavin-S and Amytracker-680 stains for Aβ plaques in APP/PS1 mouse brain.**

Coronal brain sections depicting cortical regions from wild type and APP/PS1 (male, 4 months of age) mice were stained with Thioflavin-S and Amytracker 680. Extracellular deposits of β-amyloid peptide observed as positively stained plaques (green fluorescent aggregates for thioflavin-S and red fluorescent aggregates for amytracker) in APP/PS1 mice. Scale bar = 50 μm

**Figure S4.**

**Downregulation of Akt1 in Neuro2a cells.**

(A) Neuro2a cells were transiently transfected with lipofectamine 3000 using lentiviral vector encoding scrambled RNA-Akt1 (sc-shRNA-Akt1) or shRNA against mouse Akt1 (shRNA-Akt1). After 72 hrs of transfection, cells were lysed in NP40 lysis buffer and protein concentrations were determined using BCA protein assay kit. Equal amount of cell lysates was subjected SDS-PAGE followed by immunoblotting with an antibody against Akt1. Subsequently, the immunoblot was stripped and re-probed for tubulin. (B) Densitometric analyses for Akt1 levels are normalized to tubulin. Graph represents mean – SEM from six independent experiments. Unpaired, two-tailed Mann–Whitney U test. ** indicates p<0.01.
